# Oxidative Stress: An Effective Prognostic Tool for an Early Detection of Cardiovascular Disease in Menopausal Women

**DOI:** 10.1155/2016/6157605

**Published:** 2016-03-16

**Authors:** Jyot Amrita, Mridula Mahajan, A. J. S. Bhanwer, Gurinder Mohan

**Affiliations:** ^1^Department of Biochemistry, Sri Guru Ram Das Institute of Medical Sciences and Research, Amritsar, Punjab, India; ^2^Department of Biochemistry, Government Medical College, Amritsar, Punjab, India; ^3^Department of Human Genetics, Guru Nanak Dev University, Amritsar, Punjab, India; ^4^Department of Medicine, Sri Guru Ram Das Institute of Medical Sciences and Research, Amritsar, Punjab, India

## Abstract

*Background*. Menopause, a form of reproductive aging, is marked by many hormonal variations which cause imbalance in the oxidative processes resulting in onset of endothelial dysfunction leading to cardiovascular disease (CVD). We aimed to analyze the effect of oxidative stress in an early detection of CVD in all menopausal women both normolipidemic and hyperlipidemic.* Methods and Results*. Study included 523 menopausal women (265 CVD and 258 non-CVD). They were screened for lipid profile, serum malondialdehyde (MDA), serum LDL carbonyl protein, and serum superoxide dismutase (SOD). Pearson's correlation was observed between MDA and atherogenic index of plasma (AIP) in both normolipidemic (*r* = 0.650; *p* < 0.001) and hyperlipidemic (*r* = 0.207; *p* < 0.01) CVD group as compared to non-CVD menopausal women. Significant correlation was also observed between LDL carbonyl content and AIP in normolipidemic (*r* = 0.650; *p* < 0.001) and hyperlipidemic (*r* = 0.248; *p* < 0.01) CVD menopausal women as compared to non-CVD ones.* Conclusion*. Strong correlation between atherogenic index of plasma and oxidative stress in CVD menopausal women reveals oxidative stress as an effective prognostic tool for an early detection of cardiovascular risk.

## 1. Introduction

Oxidative stress can be involved as both a primary event and a consequence of metabolic disorders, resulting in a malice cycle of oxidative stress, cardiovascular disease pathophysiology. It depends upon various environmental situations such as pollution, stress, food quality, lifestyle, and many psychosocial factors. The important role of oxidative stress in the etiology of atherosclerosis is marked by lipid alterations and oxidizability of lipoproteins. Oxidized LDL is considered to be a key event in the biological process that promotes inflammation causing endothelial injury leading to the progression of early atherosclerotic lesions [1].

Menopause, a form of reproductive aging, is marked by many hormonal variations which cause imbalance in the oxidative processes. Estrogens exert beneficial effects on endothelial dysfunction, a prerequisite of atherosclerosis by modulating lipid profile and increasing the production of nitric oxide [2]. With the decline of estrogen levels the state of menopause is manifested by many metabolic changes such as reduced glucose tolerance, dyslipidemia, redox-status imbalance, changes in body fat distribution, hypertension, endothelial dysfunction, and vascular inflammation [3, 4].

Redox-status imbalance leads to oxidative stress, a condition where reactive oxygen species (ROS)/prooxidants overcome antioxidant capacity resulting in serious cell damage. Endothelial cells and vascular smooth cells are known to be the potent sources of reactive oxygen species which are thought to be involved in the onset and development of endothelial dysfunction [5], leading to cardiovascular disease (CVD).

An increased level of oxidative stress in the body which is marked by the reduction in estrogen also depends on the concentration and chemical structure of this hormone. Specifically, at high concentration estrogen tends to have a beneficial antioxidant effect by inhibiting the 8-hydroxylation of guanine DNA bases. However, at low concentration, especially when the structure contains a catechol it has a prooxidants-like effect such as breaks in genetic material, formation of DNA adducts, and oxidation of bases [6].

Oxygen radicals can modify amino acid side chains, cleave polypeptide bonds, and make proteins more susceptible to proteolytic degradation [7] resulting in the formation of carbonyl derivatives. To counteract the detrimental effects of oxidative damage, normal living cells have an ability to control and neutralize the production of ROS by antioxidants.

Many studies have investigated menopausal CVD risk by lipid levels and oxidative stress and have compared premenopausal levels with postmenopausal levels, indicating enhanced lipid levels and oxidative stress in postmenopausal women as compared to premenopausal women [8–10]. The present study has been undertaken to specify the status of prooxidants and antioxidant in all menopausal women (natural and surgically induced menopause) with and without having CVD. Instead of comparing pre- and postmenopausal women we have considered menopausal women on both sides for comparison. Also, an effort has been made to analyze our hypothesis: whether oxidative stress does act as an effective predictor for an early detection of cardiovascular disease or not in menopausal women.

## 2. Material and Methods

### 2.1. Study Design and Selection of the Patients

A cohort of 523 menopausal women from northern Punjabi population was recruited in the present study which comprised 265 menopausal women (mean age, 44 ± 4 years) suffering from CVD. The diagnosis of heart disease was made on the basis of clinical symptoms, supportive documented ECG findings, history of the patient, and angiography as required for the disease [11] by the clinical expert. The control group comprised 258 menopausal women (mean age, 45 ± 4 years) who neither had any evidence of heart disease nor had a past history of the disease. Both the groups were matched for the age of menopause. Initially, a pilot study of 50 subjects was done to compare the levels of prooxidants, antioxidants, and lipid profile between natural menopausal women and surgically induced menopausal women. No difference in the levels was observed (data not shown). Therefore, we combined both the groups as a whole emphasizing the comparison only between CVD and non-CVD menopausal women. The subjects were classified into two groups as normolipidemics and hyperlipidemics with and without CVD. The criterion for labeling the subjects under the category of normolipidemics and hyperlipidemics was taken according to the levels recommended in National Cholesterol Education Program (NCEP) Adult Treatment Panel III guidelines (2002). Women suffering from any chronic disease, acute infections, renal disease, rheumatoid arthritis, and thyroid disorders were excluded. Women on hormonal therapy, any antioxidant supplements, and lipid lowering drugs at the time of sampling were also excluded from the study for both the groups. Informed consent and detailed clinical history including general, physical, and systemic examination were recorded for all the subjects on the pro forma prepared for the present study. The protocol was approved by the institutional ethics committee.

### 2.2. Sample Collection and Analysis

All subjects were subjected to collection of blood samples after 12 hr overnight fasting under aseptic conditions. The blood sample was then centrifuged at 3000 rpm for 15 minutes to obtain a clear serum sample. For analysis of lipid profile total serum cholesterol was estimated by the method of CHOD-PAP [16], serum triglyceride was estimated by GPO-PAP method [17], HDL-C was estimated by the method of Burstein et al. [18], VLDL-C was calculated by the formula triglycerides/5, and LDL-C was calculated by the formula of Friedewald et al. [19]. To assess lipid oxidation serum malondialdehyde (MDA) was estimated by the method of Buege and Aust [20]. To know the degree of protein oxidation LDL carbonyl content was evaluated by Yan et al. [21] and activity of superoxide dismutase (SOD), an antioxidant enzyme, was assessed by the method of Nandi and Chatterjee [22].

### 2.3. Statistical Analysis

The statistical analysis was performed using Statistical Package for Social Science program (SPSS version 16.0; SPSS Inc., Chicago, IL). All values were expressed as mean ± SD. Means for both the groups were statistically evaluated by independent Student's *t*-test. Comparison of risk factors between non-CVD and CVD menopausal women was done using chi square (*χ* ^2^) analysis. Pearson's correlation was applied to see the relationship between various risk factors. A *p* value < 0.05 was accepted as statistically significant.

## 3. Results

### 3.1. Comparison of Risk Factors between CVD and Non-CVD Menopausal Women

Table 1 enumerates the comparison of risk factors between CVD menopausal women and non-CVD menopausal women. Mean menopausal age is nearly the same in both the groups, that is, 44.95 ± 4.39 in CVD menopausal women and 45.17 ± 4.65 in non-CVD menopausal women. A statistical significant increase (*p* < 0.05) in the frequency of risk factors like hypertension and dyslipidemia was observed in CVD cases as compared to non-CVD ones, whereas obesity was found to be more in non-CVD menopausal women as compared to CVD ones (*p* < 0.05).

### 3.2. Comparison of Lipid Profile between CVD and Non-CVD Normolipidemic and Hyperlipidemic Menopausal Women

**Figure 1:**
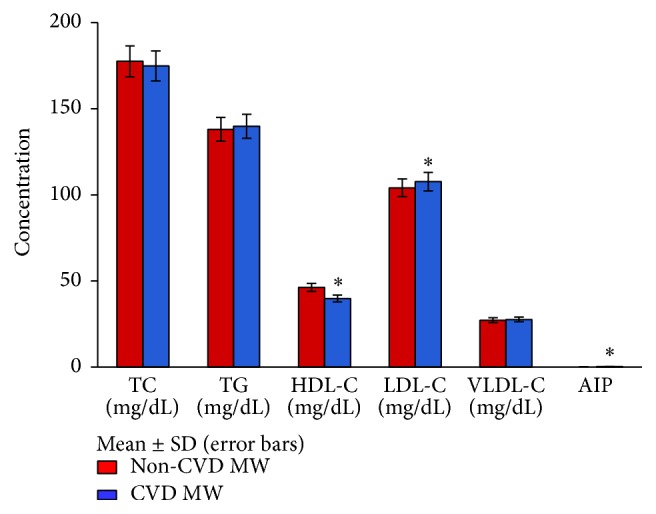
Levels of lipid profile in normolipidemic CVD and non-CVD menopausal women. TC, total cholesterol; TG, triglycerides; HDL-C, high density lipoprotein-cholesterol; LDL-C, low density lipoprotein-cholesterol; VLDL-C, very low density lipoprotein-cholesterol; AIP [log⁡(TG/HDL-C)] is taken as an index of LDL particle size; MW, menopausal women. ^*∗*^ *p* < 0.05 significant difference observed in the levels of HDL-C, LDL-C, and AIP between normolipidemic CVD and non-CVD menopausal women.* Note*. Due to small range of AIP values (mean ± SD), the bars are not visible (AIP value in non-CVD MW is 0.11 ± 0.05 and in CVD MW it is 0.19 ± 0.04).

**Figure 2:**
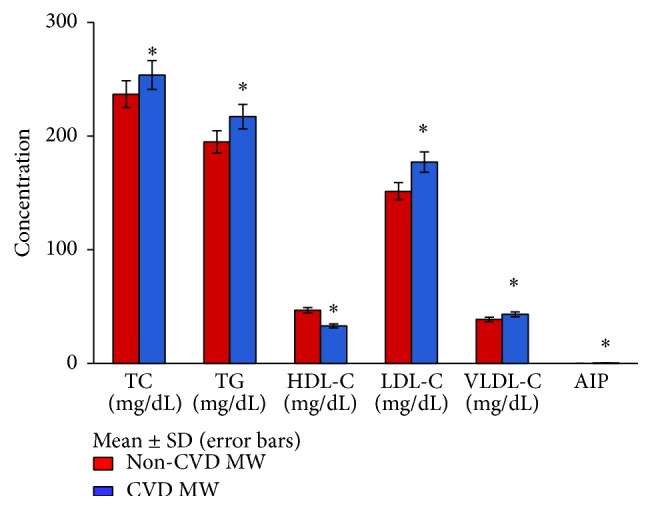
Levels of lipid profile in hyperlipidemic CVD and non-CVD menopausal women. ^*∗*^ *p* < 0.05  significant difference observed in the levels of TC, TG, HDL-C, LDL-C, VLDL-C, and AIP between hyperlipidemic CVD and non-CVD menopausal women.* Note*. Due to small range of AIP values (mean ± SD), the bars are not visible (AIP value in non-CVD MW is 0.26 ± 0.09 and in CVD MW it is 0.46 ± 0.14).

In Figure 1 levels of serum total cholesterol and serum triglycerides were nearly the same in normolipidemic CVD and non-CVD menopausal women whereas normolipidemic CVD menopausal women though in the normal range showed decreased levels of HDL-C (39.76 ± 3.91) and increased levels of LDL-C (107.60 ± 10.02) and atherogenic index of plasma (AIP) (0.19 ± 0.04) as compared to non-CVD ones (46.30 ± 5.76, 104.06 ± 11.76, and 0.11 ± 0.05), respectively. AIP [log⁡(TG/HDL-C)] is taken as an index of LDL particle size. A marked decrease (*p* < 0.05) in the levels of HDL-C and a significant rise (*p* < 0.05) in the levels of TC, TG, VLDL-C, LDL-C, and AIP have also been observed in the hyperlipidemic CVD menopausal women as compared to non-CVD ones (Figure 2). The concentration of HDL-C observed to be high in the hyperlipidemic non-CVD group explains its protective role against heart disease.

### 3.3. Comparison of Prooxidants and Antioxidant between Normolipidemic and Hyperlipidemic CVD and Non-CVD Menopausal Women

**Figure 3:**
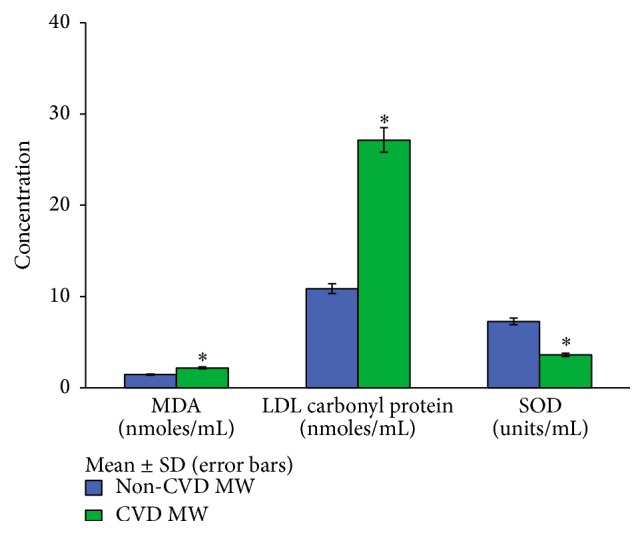
Levels of MDA, LDL carbonyl content, and SOD in normolipidemic CVD and non-CVD menopausal women. MDA, malondialdehyde; SOD, superoxide dismutase. ^*∗*^ *p* < 0.05 significant difference observed in the levels of MDA, LDL carbonyl protein, and SOD between normolipidemic CVD and non-CVD menopausal women.

**Figure 4:**
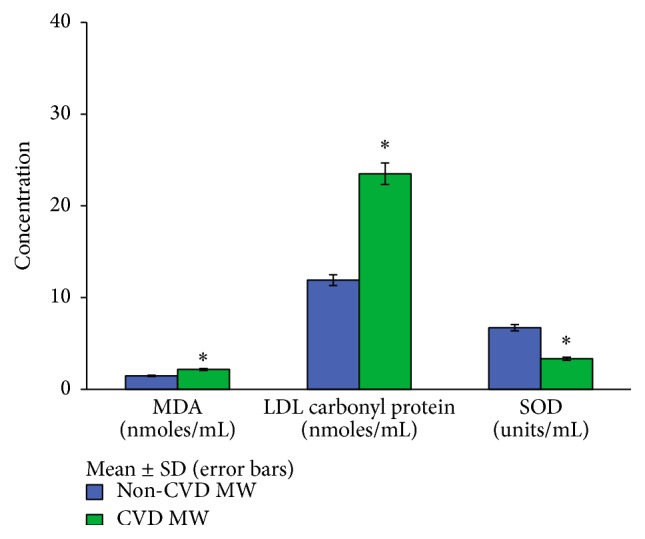
Levels of MDA, LDL carbonyl content, and SOD in hyperlipidemic CVD and non-CVD menopausal women. MDA, malondialdehyde; SOD, superoxide dismutase. ^*∗*^ *p* < 0.05 significant difference observed in the levels of MDA, LDL carbonyl protein, and SOD between hyperlipidemic CVD and non-CVD menopausal women.

Comparisons of prooxidants (MDA and LDL carbonyl content) and antioxidant (SOD) levels between normolipidemic CVD and non-CVD menopausal women and between hyperlipidemic CVD and non-CVD ones are illustrated in Figures 3 and 4, respectively. A significant increase (*p* < 0.01) in MDA (2.18 ± 0.55 versus 1.45 ± 0.40) and LDL carbonyl protein (27.15 ± 5.82 versus 10.85 ± 3.21) and a significant decrease (*p* < 0.01) in the activity of antioxidant enzyme, SOD (3.61 ± 2.73 versus 7.27 ± 2.87), used as a prognostic tool in assessing the risk of cardiovascular disease have been observed in normolipidemic CVD menopausal women as compared to non-CVD ones. For hyperlipidemics also significant differences in the levels of MDA (2.17 ± 0.62 versus 1.48 ± 0.39), LDL carbonyl protein (23.50 ± 7.30 versus 11.91 ± 3.86), and SOD (3.34 ± 2.27 versus 6.72 ± 2.69) were observed.

### 3.4. Association of Atherogenic Index of Plasma (AIP) with CVD Risk Factors

In order to ascertain any relationship between the risk factors and the LDL particle size among normolipidemic and hyperlipidemic CVD menopausal women and non-CVD ones Pearson's correlation analysis was carried out (Table 2). For most parameters among the two groups correlations are of modest to moderate magnitude (0.199–0.650 (normolipidemic group) and 0.207–0.702 (hyperlipidemic group)). A strong positive correlation has been observed between MDA and atherogenic index of plasma (AIP) in both the normolipidemic CVD group (0.650) and the hyperlipidemic CVD group (0.207). On the contrary no significant correlations have been observed between MDA and AIP in the normolipidemic non-CVD (0.118) as well as hyperlipidemic non-CVD group (0.171).

A significant negative correlation between HDL-C and AIP has been observed in all the four groups, that is, in normolipidemic non-CVD (−0.800), normolipidemic CVD (−0.799), hyperlipidemic non-CVD (−0.632), and hyperlipidemic CVD (−0.813). However, a significant positive correlation of TG with AIP was found in all the four groups, that is, in normolipidemic non-CVD (0.356), normolipidemic CVD (0.439), hyperlipidemic non-CVD (0.666), and hyperlipidemic CVD (0.702), whereas significant association of LDL-C with AIP was only found in normolipidemic CVD group (0.310) and in hyperlipidemic CVD group (0.248).

With regard to the association of LDL carbonyl content and AIP, positive significant correlation was observed in normolipidemic CVD group (0.650) and in hyperlipidemic CVD group (0.385). Conversely, no such significant correlation was observed in non-CVD group of both normolipidemia and hyperlipidemia.

## 4. Discussion

Cardiovascular disease (CVD) and its associated unfavorable complications are the major cause of morbidity and mortality especially in women at menopausal age despite recent advances in diagnostic facilities and treatment modalities. Role of hyperlipidemia in the pathophysiology of atherosclerotic CVD as a risk factor is clear. However, there are many additional mechanisms such as acute inflammatory response [23] which starts with stimulated endothelium [24] and renders these pathological changes to chronic disease like atherosclerosis. This activated endothelium produces cell type-specific agonists for adherent monocytes, neutrophils, or lymphocytes [25]. Mostly, every vascular cell type forms lipid oxidation products which participate in these processes [26]. Estrogens are antiatherogenic substances which reduce endothelial LDL-C incorporation; therefore, their deficiency in menopausal women increases endothelial dysfunction and formation of prooxidants.

Lipid peroxidation is believed to be involved in the peroxidative modification of low density lipoproteins (LDL). Oxidative (modified) LDLs have diverse and potent effects throughout the inflammatory response and play prominent roles in atherosclerotic changes. Increased production of these reactive oxygen species (ROS) like superoxide ion mediates various signaling pathways that underline vascular inflammation in atherogenesis [27]. These partial changes may help to explain the frequent occurrence of the disease in normolipidemics [28, 29], as we also observed in our study. To the best of our knowledge no study so far has identified the relationship between prooxidants and antioxidants in hyperlipidemic and normolipidemic menopausal women with and without CVD. So, we tried to focus on this relationship.

From our investigations we observed that 37% of noteworthy population of women had normal lipid profile but still they suffered from heart disease. This reflects the weak predictive influence of the lipid levels. These women are thus at increased risk of heart disease in the absence of better risk indicators which are needed to be explored.

Accordingly, evaluation of oxidant-antioxidant profile may have an added advantage over the estimation of lipid profile. Our normolipidemic CVD group revealed that lipid profile could not competently distinguish it from the subjects of non-CVD group. This is also supported by Stringer et al. [28] who observed marginal increase of cholesterol in heart patients as compared to controls and a weak correlation of TC with lipid peroxides.

In our population of normolipidemic and hyperlipidemic menopausal women TC showed no correlation with atherogenic index of plasma. Atherogenic index of plasma (AIP) defined as log⁡TG/HDL-C is taken as an index of LDL particle size which indicates a balance between the actual concentration of plasma TG and HDL-C which predetermine the flux of newly formed cholesteryl esters by LCAT towards atherogenic LDLs or beneficial HDLs [30]. Persons with a prevalence of small dense particles are classified as having LDL phenotype pattern B compared to pattern A which is characterized by large LDL particle predominance. Family studies have suggested that pattern B is inherited as a dominant single trait with a population frequency of 25% [31]. This demonstrates the heterogenic nature of LDL. Several lines of evidence have suggested predominance of small dense LDL particles in coronary artery disease [32, 33] rather than the presence of hypercholesterolemia. Decreased level of HDL-C of normolipidemic menopausal women of our environment may be due to the fact of their less active and sedentary lifestyle.

Malondialdehyde (MDA) which is one of the products of lipid peroxidation has been the most extensively studied marker. Its increased level marks the index of assessing oxidative stress. Accordingly, we observed an increase in the levels of MDA in both hyperlipidemic and normolipidemic menopausal women which indicated the degree of oxidation in heart disease (Figures 3 and 4).

But appraisal of lipid oxidation may not always be informative for CAD risk assessment. Therefore, we also attempted to study LDL oxidation in terms of its protein content. Unlike lipid oxidation, protein oxidation does not have the features of chain reactions. Enhanced levels of carbonyl derivatives are formed when proteins become more susceptible to proteolytic degradation on exposure to oxygen radicals. Protein carbonyl content is actually the broadest indicator and by far the most commonly used marker of protein oxidation. In the light of this fact we estimated carbonyl content of LDL as an index of protein oxidation. Significant increase in the levels of LDL carbonyl content was observed in both normolipidemic and hyperlipidemic menopausal women. Interestingly, levels of carbonyl content were found to be more in normolipidemic ones as compared to hyperlipidemic menopausal women with CVD. It means that some oxidative events initiate even when LDL levels are normal.

Extracellular SOD (EC-SOD) is a secretary glycoprotein whose levels are found high in blood vessels so as to suppress oxidative stress under normal conditions. Our findings corroborate this theory as we observed low levels of SOD in both normolipidemic and hyperlipidemic CVD menopausal women indicating free radical generation and simultaneously decreased antioxidant production.

A new aspect gained from the present investigations indicated increased value of AIP in normolipidemic CVD menopausal women as compared to non-CVD ones. Moreover strong correlation of LDL oxidation (MDA and carbonyl content) with AIP in normolipidemic CVD menopausal women is in line with the fact that oxidation is due to the presence of small dense LDL particles and that oxidative processes seem to start earlier, even when LDL and TC levels are normal. This is also in concordance with another study [34]. Guerci et al. in their study revealed that LDL particles of normolipidemic type 2 diabetic women were more susceptible to oxidation leading to risk of cardiovascular events [35]. Same scenario was also observed in hyperlipidemic menopausal women which clearly indicated that oxidative stress is an early event in the evolution of hyperlipidemia leading to cardiovascular disease. This is also supported by the study of Augusti et al., 2012 [36].

In conclusion, strong correlation observed between atherogenic index of plasma (AIP) and oxidative stress in normolipidemics demonstrates that oxidative events initiate even when lipid levels are normal and moreover normolipidemic menopausal women are also equally susceptible to heart risk irrespective of the lipid status. It also visualizes that redox-status imbalance can be considered as an effective prognostic tool for an early detection of cardiovascular risk.

*Additional Points*. In order to modify risks of CVD in women, intervention with regard to keeping a balance between prooxidants and antioxidant status at the earlier stages of menopause is imperative.

## Figures and Tables

**Table 1:** Frequency of risk factors in menopausal women (with and without CVD).

Risk factors	Group	
	Non-CVD menopausal women Mean age (45 ± 4 years)	CVD menopausal women Mean age (44 ± 4 years)
Hypertension (BP ≥ 140/90) (US JNCVII, 2003) [12]	0.46	0.69^*∗*^

Dyslipidemia (ATP III guidelines, 2002) [13]	0.45	0.62^*∗*^

Diabetes (B sugar (F) ≥ 110 mg/dL) (WHO, 2000) [14]	0.24	0.26

Obesity (BMI ≥ 25 kg/m^2^) (WHO, 2000) [15]	0.62	0.53^*∗*^

^*∗*^ *p* < 0.05 was considered statistically significant.

Comparison of frequency of risk factors between non-CVD menopausal women and CVD menopausal women was done using chi square (*χ* ^2^) analysis.

**Table 2:** Correlation matrix (bivariate) of various risk factors among normolipidemic and hyperlipidemic menopausal women with and without CVD.

	AIP [log⁡(TG/HDL-C)]							
Variables	Normolipidemic non-CVD menopausal women		Normolipidemic CVD menopausal women		Hyperlipidemic non-CVD menopausal women		Hyperlipidemic CVD menopausal women	
	*r*	*p* value	*r*	*p* value	*r*	*p* value	*r*	*p* value
TC	−0.179	<0.05	−0.068	NS	0.013	NS	0.243	<0.01
TG	0.356	<0.01	0.439	<0.001	0.666	<0.001	0.702	<0.001
VLDL-C	0.324	<0.01	0.396	<0.01	0.680	<0.001	0.700	<0.001
LDL-C	0.135	**NS**	0.310	**<0.01**	0.048	**NS**	0.248	**<0.01**
HDL-C	−0.800	<0.001	−0.799	<0.001	−0.632	<0.001	−0.813	<0.001
MDA	0.118	**NS**	0.199	**<0.05**	0.171	**NS**	0.207	**<0.01**
LDL cbpr.	−0.024	**NS**	0.650	**<0.001**	0.046	**NS**	0.385	**<0.001**
SOD	−0.121	NS	0.032	NS	0.026	NS	0.031	NS

AIP is atherogenic index of plasma taken as an index of LDL particle size.

**p** < 0.05 is significant, **p** < 0.01 is highly significant, and **p** < 0.001 is extremely significant.
